# Limited Evidence to Fully Determine the Implementation of Evidence‐Based Practice by Healthcare Providers in Africa: A Systematic Review and Meta‐Analysis

**DOI:** 10.1111/jebm.70032

**Published:** 2025-05-15

**Authors:** Feleke H. Astawesegn, Kedir Y. Ahmed, Subash Thapa, Shakeel Mahmood, Anayochukwu Anyasodor, M. Mamun Huda, Setognal B. Aychilihum, Utpal K. Modal, Allen G. Ross

**Affiliations:** ^1^ Rural Health Research Institute (RHRI) Charles Sturt University Orange New South Wales Australia; ^2^ Hawassa University, Hawassa Ethiopia

**Keywords:** Africa, clinical outcomes, evidence‐based medicine, evidence‐based practice, healthcare providers

## Abstract

**Aim:**

Implementing evidence‐based practice (EBP) is a complex process requiring healthcare providers to integrate evidence‐based medicine (EBM) into clinical practice, ultimately improving clinical outcomes. This systematic review examined the sources of information for EBP, analyzed the extent of EBP implementation by healthcare providers, and explored the factors influencing EBP in Africa.

**Methods:**

We identified articles published between January 1992 and March 2024 by searching Cumulative Index to Nursing and Allied Health Literature (CINAHL), EMbase, PubMed, and Scopus databases. The pooled effect sizes for the prevalence of EBP and odds ratios (ORs) were estimated using random‐ and fixed‐effects models as appropriate. For the qualitative component of the study, we performed a thematic analysis and subsequently integrated and interpreted findings from both the quantitative and qualitative analyses.

**Results:**

Thirty‐three studies were included in this review, involving 9722 healthcare providers: 60.3% nurses, 15.9% physicians, and 15.4% midwives. Our findings revealed a lack of detailed information on how healthcare providers utilized different forms of EBM to inform EBP and clinical outcomes in Africa. Self‐reported EBP was 57.3% among nurses and 37.3% among physicians. Nigeria had the highest self‐reported EBP (75.2%), whereas Egypt had the lowest (18.9%). Common sources of information reported for EBP were PubMed, UpToDate, the Cochrane Library, clinical guidelines, and training programs. Factors associated with EBP included knowledge of EBP (OR = 2.13, 95% confidence interval [CI]: 1.83–2.47), positive attitude toward EBP (OR = 1.95, 95% CI: 1.76–2.15), and having EBM training (OR = 3.08, 95% CI: 2.08–4.57), and a managerial role (OR = 2.16, 95% CI: 1.37–3.41). The availability of guidelines (OR = 1.88, 95% CI: 1.5–2.37) and internet access (OR = 1.90, 95% CI: 1.54–2.34) were also found to increase EBP. Our qualitative analysis identified common barriers to EBP, including a lack of support, resistance to change, poor communication, and failure to integrate EBP courses into the continuing education curricula.

**Conclusion:**

This systematic review found limited information on the sources of EBM, how it was delivered, and its frequency of use in clinical practice. Thus, the correlation between EBM, EBP, and clinical outcomes was not fully transparent. Further studies are required to examine the medical conditions addressed within providers’ scopes of practice, the types of evidence utilized, the frequency and consistency of EBP implementation, and its effect on enhancing patient outcomes.

**Abbreviations**
CDS: clinical decision support
CI: confidence interval
CINAHL: Cumulative Index to Nursing and Allied Health Literature
CPD: continuous professional development
EBM: evidence‐based medicine
EBP: evidence‐based practice
GIM: Global Index Medicus
MMAT: Mixed‐Method Assessment Tool
OR: odds ratio
PE: predictor effect
PRISMA: Preferred Reporting Item for Systematic Reviews and Meta‐Analysis
PROSPERO: International Prospective Register of Systematic Reviews
RR: relative risk

## 1 Introduction

Evidence‐based practice (EBP) involves the integration of clinical expertise, patient values, and the best evidence into the decision‐making process for patient care [1, 2]. Building on evidence‐based medicine (EBM), EBP provides a shared framework for problem‐solving, process improvement, communication, and understanding among stakeholders, such as health practitioners, patients, families, and carers [3, 4]. EBP not only reduces variations in clinical practices but also promotes best practices, lowers costs, improves healthcare quality, and increases patient satisfaction [2, 5, 6, 7, 8, 9].

Despite the benefits, implementing EBP in healthcare is challenging due to the complexity of the sector, the diversity of healthcare practitioners (e.g., doctors, nurses, midwives, pharmacists, and podiatrists), specialties (e.g., internists, surgeons, pathologists, radiologists, and psychiatrists), patient needs, the nature and quality of the evidence, the specific context where the evidence needs to be applied, and the necessity for robust data collection and analysis systems to evaluate its impact on clinical outcomes [10, 11]. Previously, EBP has been applied across various fields to guide clinical practices. For example, in medicine, EBP was used to inform specific practices such as spine surgery [12], phototherapy for psoriasis [13], chronic pancreatitis surgery [14], and screening for colorectal cancer [15, 16]. In nursing, EBP was used for acute stroke care [17] and prevention of surgical site infections [18]. In midwifery, EBP promotes a healthy lifestyle during pregnancy [19] and prevents post‐caesarean infections [20]. Pharmacists use EBP to manage drug–drug interactions [21] and ensure medication safety [22].

Healthcare providers must continuously update their knowledge and skills with the latest research to ensure personalized and effective patient care. Continuous professional development (CPD) helps to update and enhance providers’ knowledge, skills, and performance to deliver appropriate and safe healthcare [23, 24]. CPD includes formal courses, workshops, and self‐directed learning, whereas tailored evidence‐based guidelines help standardize care across diverse healthcare practices. Globally, various platforms and tools have been also developed to support the CPD process, including online databases like the Cochrane Library and mobile apps such as UpToDate, Medscape, and Skyscape for the latest medical information [25, 26]. Clinical decision support (CDS) systems in electronic health records also provide real‐time decision‐making assistance [27].

In Africa, there is a growing recognition of the importance of evidence‐based healthcare, demonstrated by various recent initiatives [28, 29]. However, previous individual studies have shown that healthcare providers frequently underutilize available evidence [30, 31, 32]. Contributing factors included a lack of internet access [33], academic qualifications [34], work experience [33], limited time, low awareness [30, 34], limited critical appraisal skills [30], and lack of motivation due to lack of incentive [35]. Although these individual studies are important and deserve recognition, no systematic review has pooled the overall prevalence of EBP and its associated factors in Africa. This systematic review was conducted to examine the sources of information for EBP, to understand the extent to which healthcare providers implement EBP within their scope of practice, and the factors associated with EBP in Africa.

## 2 Methods

The Preferred Reporting Item for Systematic Reviews and Meta‐Analysis (PRISMA) was followed to report the study (Table S1). The review was registered on the International Prospective Register of Systematic Reviews (PROSPERO) with a unique identifying number CRD42024517704.

### 2.1 Inclusion and Exclusion Criteria

Our inclusion criteria included: [1] studies that used quantitative, qualitative, and mixed methods; [2] studies conducted on healthcare providers (e.g., physicians, nurses, dentists, pharmacists, physiotherapists, radiographers, occupational therapists, community health officers, and laboratory workers); [3] articles that reported the magnitude of EBP and statistically significant factors; [4] published from January 1992 to March 05, 2024; [5] studies written in the English language; and [6] studies conducted in Africa. Exclusion criteria included: [1] systematic reviews; [2] studies conducted on undergraduate students from any healthcare discipline; and [3] articles about EBP theory, framework development, validation, and evaluating the impact of various evidence on a specific disorder were also excluded.

### 2.2 Information Sources and Search Strategies

The literature search was conducted in PubMed, Embase, Cumulative Index to Nursing and Allied Health Literature (CINAHL), Scopus, Global Index Medicus (GIM), and Google Scholar to identify studies published from January 1992 to March 2024 (1992 was the year in which EBM was first introduced in the literature [36]). The search strategy was built on three themes (1) EBP (e.g., “EBM” OR “evidence‐informed practice” OR “evidence‐based healthcare”) AND (2) healthcare providers AND (3) setting (all African countries). The detailed search strategy is provided in Table S2.

### 2.3 Study Selection and Data Extraction

All identified citations were collected and uploaded into the reference management software EndNote (version X9), and duplicates were removed. After removing duplicates, we reviewed the titles and abstracts of the remaining records and removed those that did not meet the inclusion and exclusion criteria. The remaining articles underwent a full‐text review, and reasons for excluding studies were documented. Finally, data were extracted using an Excel template across the following domains: (i) aim of the study; (ii) country; (iii) year of study; (iv) year of publication; (v) setting/clinical area; (vi) profession type; (vii) study type; (viii) sample size; (ix) proportion of EBP; (x) reported odds ratio (OR) or relative risk (RR) and its 95% confidence interval (CI) estimates of significant factors from quantitative studies; and (XI) the phenomenon under investigation from qualitative studies. All data were extracted and verified by two reviewers (FH and KA), and discrepancies between the reviewers were resolved through discussion. This process of screening and data extraction ensured the preparation dataset for analysis.

### 2.4 Assessment of Methodological Quality

The methodological quality of the included studies was evaluated using the Mixed‐Method Assessment Tool (MMAT) version 2018 [37]. The MMAT provides a unique tool to assess the methodological quality of quantitative, qualitative, and mixed‐methods studies. On the basis of a total score, studies are put into three categories: low quality (0–3), moderate quality (4 and 5), and high quality (6 and 7). Quality scores for each study were presented in Table S3.

### 2.5 Study Outcomes

The main outcome of this review was EBP, defined as a problem‐solving approach to clinical decision‐making that integrates the best available evidence with clinicians’ expertise and patients’ personal preferences and values [1, 38]. This involves asking questions, acquiring the best evidence, appraising the evidence, applying the findings to clinical practice, and evaluating the outcomes of change [39, 40]. The studies included in this review assessed providers’ EBP through composite variables, using a structured, self‐administered questionnaire that had been pre‐tested and adapted from various literature sources [41, 42, 43, 44, 45, 46, 47]. Accordingly, providers’ EBP was calculated by dividing the number of healthcare providers with good EBP (as reported in the studies) by the total number of healthcare providers in the study multiplied by 100. Another primary outcome of this review was individual, professional, and organizational factors that showed significant associations with EBP in multiple logistic or linear regression analyses.

### 2.6 Data Analysis

After importing quantitative studies into the R environment for metanalysis, the pooled proportion of EBP along with 95% CIs was estimated. Following this, subgroup analyses were conducted on the basis of the country of study, study type, publication year, and providers’ profession to examine the level of EBP across different contexts. Additionally, to understand the influence of different explanatory variables with EBP, we conducted a meta‐analysis for each potential explanatory factor, represented as the predictor effect (PE) for EBP and its standard error (sePE), derived from available adjusted ORs and their corresponding 95% CIs. The pooled effects of these factors were reported using ORs and 95% CIs, with forest plots generated to present the observed variation across studies. A narrative synthesis was conducted for factors identified in two or fewer studies as well as for those where their effect size was measured using the beta coefficient.

Heterogeneity between studies was evaluated using both *I*‐squared statistics and Cochran's *Q* test. The *I* ^2^ statistic describes the percentage of total variation across studies attributable to heterogeneity observed [48]. Accordingly, an *I* ^2^ statistic of 25% or lower suggests negligible heterogeneity, whereas 26%–50% indicates low heterogeneity, 51%–75% denotes moderate heterogeneity, and 75% or higher indicates significant heterogeneity [48]. On the basis of the heterogeneity level between studies, a fixed‐effect or random‐effect model (where applicable) was chosen using Cochran's *Q* test, with a significance level of *p* < 0.05. Additionally, Egger's tests and funnel plot were used to evaluate publication bias within the studies analyzed in the meta‐analysis.

For qualitative synthesis, findings from the results section of the qualitative studies, including the qualitative part of the mixed‐methods study, were extracted and underwent thematic analysis. This process involved familiarization with the data by reading and re‐reading the original studies, generating codes, and categorizing on the basis of their similarities [49]. Subsequently, these categories were further organized into four overarching themes (healthcare provider, healthcare organization, education, and evidence‐related factors) and reporting findings in the result narrative synthesis. Finally, integration and interpretation of findings from both the quantitative and qualitative analysis were performed [50]. All meta‐analyses were conducted using R version 4.3.3 using meta‐analysis packages “metagen,” “tidyverse,” and “meta” as appropriate.

## 3 Results

### 3.1 Study Selection

**FIGURE 1:**
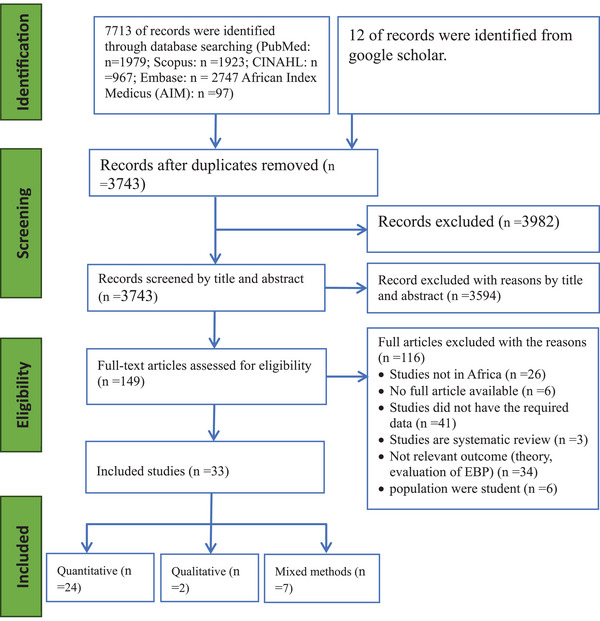
Flow diagram illustrating the selection process of articles utilized in this systematic review [51]. CINAHL, Cumulative Index to Nursing and Allied Health Literature; EBP, evidence‐based practice.

Our electronic searches retrieved a total of 7725 articles, and 3982 duplicate records were removed. Of the 3743 articles screened for eligibility, 3594 were excluded by their title and abstract evaluation. Out of the remaining 149 records, 116 studies were removed at full‐text review due to a lack of specific focus on the EBP, absence of the required data, and student study population. Finally, a total of 33 studies were included in this review (Figure 1).

### 3.2 Study Characteristics

Twenty‐four studies used quantitative methods (72.7%), followed by seven mixed methods studies (21.2%). Most of these studies were conducted in Ethiopia (63.6%), followed by Nigeria (6.6%) and Kenya (6.6%). Overall, the number of publications on EPB has been increasing over the past decade, starting with the first publication in 2010 [52] and the most recent ones published in 2024 [52, 53, 54]. All studies were conducted in a hospital‐based setting using a cross‐sectional study design. The studies included 9722 healthcare providers (9565 for quantitative and 157 for qualitative). Of the healthcare providers included in this review, nurses account for 5859 (60.26%), physicians for 1550 (15.94%), midwives for 1500 (15.42%), and other professionals such as pharmacists, medical laboratory staff, dentists, and radiographers constituted 813 (8.38%).

Moreover, the studies have involved participants from various departments, including the Medical Department [32, 33, 55, 56, 57, 58, 59, 60, 61], Surgical Department [32, 33, 55, 56, 57, 58, 59, 60, 61], Emergency Department [55, 57, 58, 59, 60, 62, 63], Critical Care Department [33, 55, 58, 59, 62, 63, 64], Pediatrics Department [32, 33, 55, 57, 58, 59, 60, 63], Outpatient Department [33, 55, 57, 58, 59, 62], Operation Room [58, 60, 62] Inpatient Ward [32, 57, 59, 60, 62], and other areas such as Dermatology, Oncology, Pathology, Radiology, and Laboratory [32, 64, 65, 66]. Details of the characteristics of these studies are provided in Table S4.

### 3.3 Evidence‐Based Practice

**FIGURE 2:**
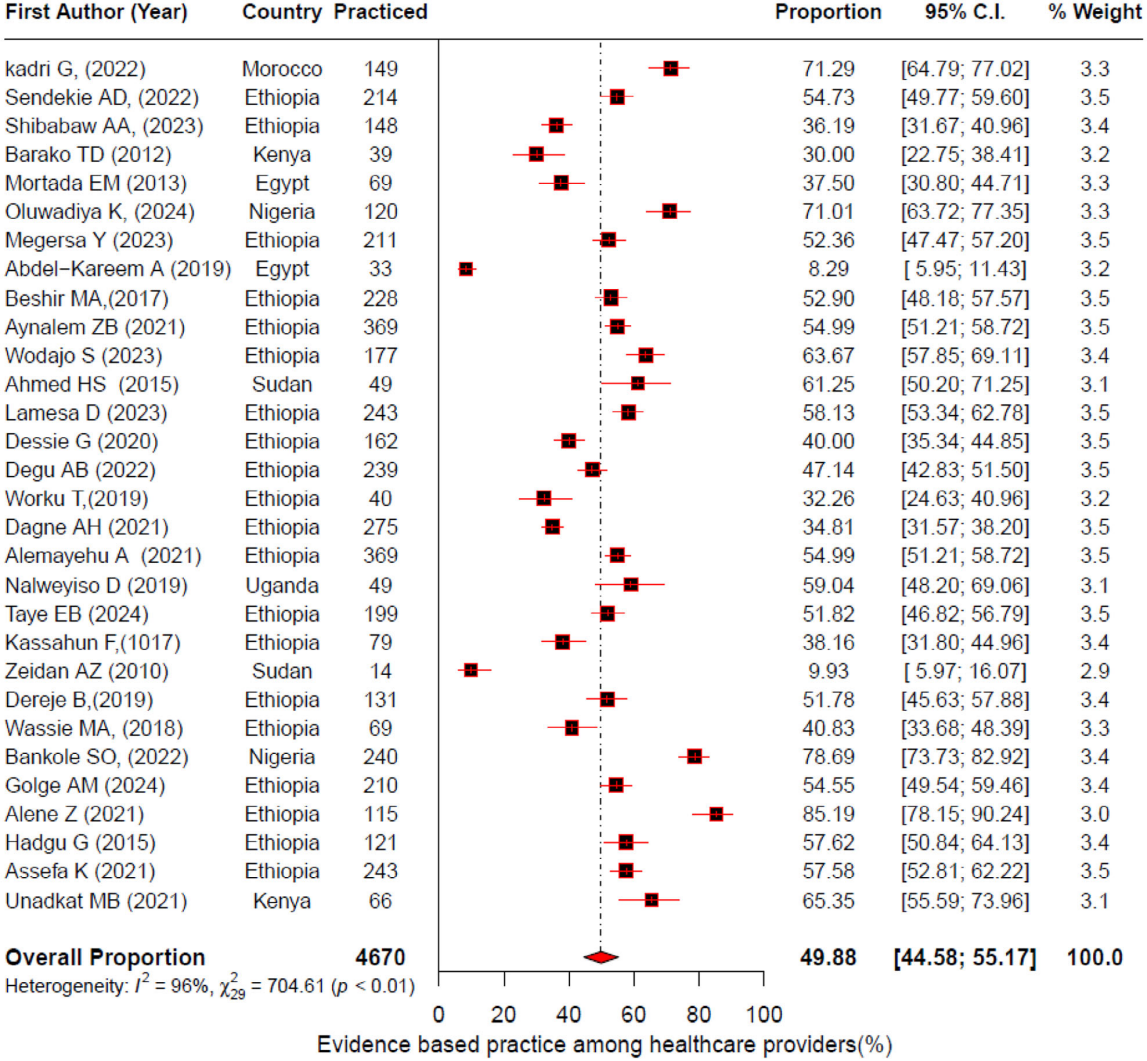
Forest plot to estimate the pooled proportion of EBP among healthcare providers with 95% confidence interval (CI) on the basis of the random effects model.

Thirty studies quantitatively investigated the level of EBP among healthcare providers in Africa [31, 32, 33, 51, 52, 53, 54, 55, 56, 57, 58, 59, 60, 62, 63, 65, 66, 67, 68, 69, 70, 71, 72, 73, 74, 75, 76, 77, 78, 79]. The overall pooled proportion of EBP among healthcare providers was 49.88% (95% CI: 44.58%, 55.17%) with higher heterogeneity among studies (*I* ^2^ = 96%, *p* value < 0.01) (Figure 2). Our pooled analysis to examine the knowledge and attitude of healthcare providers for EBP showed that 56% (95% CI: 47%, 64%) had good knowledge of EBP and 68.0% (95% CI: 59.0%, 76.0%) had a positive attitude toward EBP, with high heterogeneity among studies (Figure S1).

### 3.4 Subgroup Analysis

Our subgroup analysis showed that the proportion of EBP varied markedly across countries. Nigeria and Morocco had the highest estimated proportions, 75% (95% CI: 67%–82%) and 71.3% (95% CI: 64.8%–77%), respectively, whereas Egypt had the lowest 18.9% (95% CI: 3.5%–59.9%). The proportion of EBP among physicians and nurses was 37 % (95% CI: 16; 64.7%) (*I* ^2^ = 98%) and 57 % (95% CI: 51.7%–62.8%) (*I* ^2^ = 93%), respectively. The high study heterogeneity remained unchanged after subgroup analysis; hence, the random effects model estimate was reported (Table 1).

**TABLE 1:** The pooled proportion of evidence‐based practice (EBP), 95% confidence interval (CI), and heterogeneity estimate for the subgroup analysis.

				Heterogeneity	
Subgroup variables	No of studies	Proportion (%) (95% CI)	Weight	*I* ^2^ (%)	*p* value
**Country**					
Ethiopia	20	51.00 (46.62, 55.37)	68.1	93%	*p* < 0.01
Morocco	1	71.29 (64.79, 77.02)	3.3		
Kenya	2	47.28 (17.35, 79.30)	6.3	96%	*p* < 0.01
Egypt	2	18.93 (3.53, 59.87)	6.6	98%	*p* < 0.01
Nigeria	2	75.25 (67.05, 81.96)	6.6	71%	*p* < 0.06
Sudan	2	29.55 (2.99, 85.08)	5.9	98%	*p* < 0.01
Uganda	1	59.04 (48.20, 69.06)	3.1		
**Healthcare providers type**					
Dentists	1	71.29 (64.79, 77.02)	3.3		
Nurses	12	57.33 (51.67, 62.80)	40.6	93%	*p* < 0.01
Physicians	6	37.34 (16.21, 64.74)	18.9	98%	*p* < 0.01
Radiographers	1	59.04 (48.20, 69.06)	3.1		
Midwives	1	51.82 (46.82, 56.79)	3.5		
Medical laboratory	1	40.83 (33.68, 48.39)	3.3		
Mixed	8	44.05 (36.53, 51.85)	27.3	94%	*p* < 0.01
**Study type**					
Quantitative	24	49.45 (43.09, 55.82)	80.0	97%	*p* < 0.01
Mixed methods	6	51.56 (43.95, 59.08)	20.0	88%	*p* < 0.01
**Sample size**					
<300	15	51.47 (42.03, 60.81)	48.4	95%	*p* < 0.01
≥300	15	48.35 (41.66, 55.10)	51.6	97%	*p* < 0.01
**Year of publication**					
>2015	25	52.31 (46.73, 57.83)	84.2	96%	*p* < 0.01
≤2015	5	36.78 (21.43, 55.37)	15.8	95%	*p* < 0.01
**Overall**	30	49.88 (44.58, 55.17)	100.0	96%	*p *< 0.01

### 3.5 Factors Associated With EBP

**FIGURE 3:**
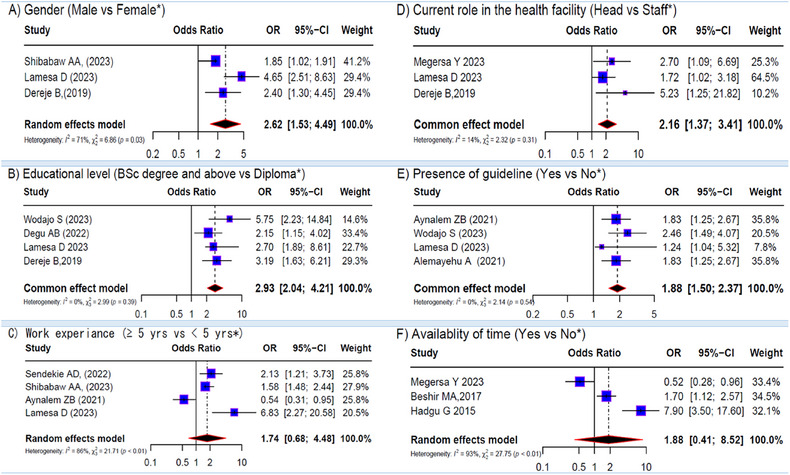
Forest plots depicting the pooled effects for the significant findings from all studies. (A) gender; (B) educational level; (C) work experience; (D) current role; (E) presence of guideline; (F) availability of time (reference group*). CI, confidence interval.

**FIGURE 4:**
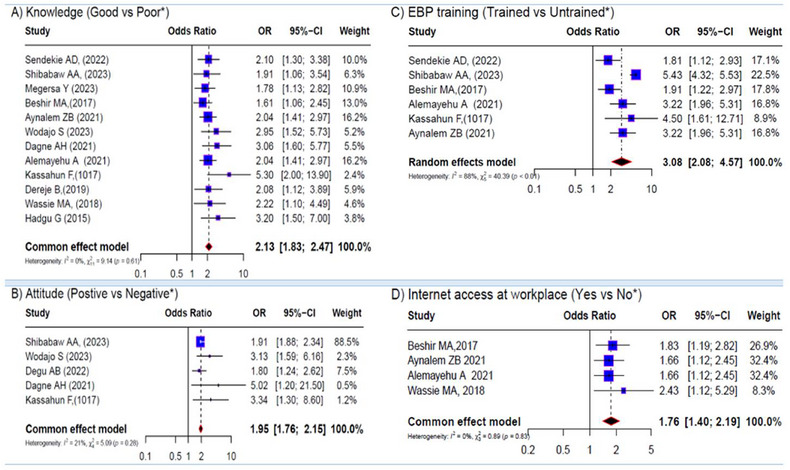
Forest plots depicting the pooled effects for the significant findings from all studies. (A) knowledge; (B) attitude; (C) EBP training; (D) internet access (reference group*). CI, confidence interval.

A total of 10 variables were eligible for meta‐analyses of factors associated with EBP. Except for work experience and time availability, the pooled effects of all other variables (gender, educational level, current role, EBP training, knowledge, attitude toward EBP, the presence of guidelines, and internet access at the workplace) were found to be significant. The forest plots in Figures 3 and 4 depict the overall effect sizes of these factors.

A pooled analysis of three studies indicated that male providers were positively associated with EBP (OR = 2.62; 95% CI: 1.53, 4.49), with a moderate level of heterogeneity among studies (*I* ^2^ = 71%; *p* = 0.03) (Figure 3A). Additionally, healthcare providers holding a bachelor's degree or higher were more likely to implement EBP (OR = 2.93; 95% CI: 2.04–4.21), with no observed heterogeneity (*I* ^2^ = 0.0%, *p* = 0.39) (Figure 3B). The review identified three eligible studies [58, 59, 63] encompassing 1074 healthcare providers, which examined the effect of healthcare providers’ roles within a facility on EBP. It was found that healthcare providers in management positions had significantly higher odds of EBP compared to regular staff (OR = 2.16, 95% CI 1.37–3.41) (Figure 3D). Similarly, the meta‐analysis using a random effects model demonstrated that guideline availability was a significant predictor of EBP (OR 1.88, 95% CI 1.5–2.37) (Figure 3E).

Good knowledge was identified as a significant factor for EBP in 14 studies [33, 35, 52, 55, 59, 63, 65, 67, 68, 69, 71, 72, 75, 78]. However, two studies [52, 68] were excluded from the meta‐analysis due to differing analysis methods, primarily their use of linear regression. Consequently, the pooled estimate from twelve studies [33, 35, 55, 59, 63, 65, 67, 69, 71, 72, 75, 78] involving 4883 healthcare providers showed that those with good knowledge of EBP were 2.13 times more likely to use evidence in their clinical decisions compared to those with poor knowledge (OR = 2.13, 95% CI = 1.83–2.47) (Figure 4A). Similarly, 5 studies [62, 69, 72, 74, 75] involving 2191 healthcare providers were included to assess the effect of providers’ attitudes. The fixed effect model revealed a significant association between providers’ attitudes and EBP (OR = 1.95, 95% CI = 1.76–2.15) (Figure 4B). Moreover, the meta‐analysis based on six studies [33, 55, 67, 69, 71, 75] indicated that EBP‐trained healthcare providers were more likely to implement EBP than their counterparts (OR, 3.08; 95% CI: 2.08–4.57), with higher heterogeneity (*I* ^2^ = 88%, *p* < 0.01). Additionally, a pooled estimate between internet access at the workplace and EBP showed a significant association (OR 1.76, 95% CI 1.40–2.19) (Figure 4D).

Finally, there were additional significant factors related to EBP that we did not include in our meta‐analysis due to the limited number of studies and the variations in statistical regression methods. These factors, which have a positive effect on EBP, include older age [74, 78], single marital status [55, 65], effective nurse‐patient communication [33, 55], good EBP culture [33, 65], supportive administration [62, 78], and colleagues [60], participating in conferences or seminars [53, 75], attending CPD [54], and self‐efficacy [52, 74]. Conversely, a higher workload [73] and a lack of research skills [56, 60, 63] were identified as hindering factors (Table S4).

### 3.6 Results From Qualitative Analysis

Eight studies (including the qualitative data presented in the mixed‐methods study) were included in the qualitative synthesis [32, 35, 56, 62, 63, 64, 79, 80]. Findings were categorized into four different themes [1] healthcare providers, [2] healthcare organization factors, [3] education factors, and [4] evidence‐related factors (Table S5).

Healthcare providers’ factors: Several studies [35, 62, 64, 80] highlighted that providers’ knowledge and skill gaps had a detrimental effect on EBP implementation. Similarly, time constraints to search for evidence and increased workloads attributed to staff shortages were consistently identified as major barriers to EBP across multiple studies [32, 35, 56, 64, 79, 80]. Findings from qualitative studies also indicated that the lack of EBP training or workshops was noted to affect providers’ engagement in EBP [35, 62, 64, 79]. Furthermore, there was evidence suggesting that a lack of motivation [35, 63], resistance to change [62, 63, 79], and poor communication [63, 79] also acted as barriers to EBP.

Healthcare organizations factors: Frequently cited organizational‐level reasons for the non‐use of evidence in clinical decisions included insufficient support from supervisors or managers [35, 62, 63, 79] and poor information technology services [81]. Moreover, studies highlighted insufficient staffing [56, 80], inadequate computer facilities [35, 62, 63, 79], absence of guidelines [35, 56, 63, 64, 79], and lack of libraries and internet access [35, 63, 64, 79] as a major barrier to EBP. Note that having established training centers and experience‐sharing between hospitals were described as enabling healthcare factors in translating evidence to practice [63].

Educational and evidence‐related factors: One study pointed out a lack of educational opportunities to advance providers’ careers and not incorporating the concepts of EBP in various academic program curricula as barriers to EBP [81]. Furthermore, four studies [35, 62, 63, 79] noted that insufficient research articles/literature/scientific documents for EBP were identified as a challenge.

### 3.7 Sources of Information

Most of the studies reported electronic databases and websites such as PubMed/Medline [32, 54, 60, 61, 62, 68, 81], UpToDate [32, 60, 61, 62, 81], Cochrane Library [32, 54, 60, 61, 62, 67, 68, 72], Medscape [61, 62], HINARY [32, 60], ClinicalKey [32, 61], and Google Scholar [32, 60, 68] along with national guidelines [52, 54, 58, 59, 62, 65, 81], hospital protocols [58, 59, 65], and WHO guidelines [67, 72, 81] as sources of information for EBP. Moreover, they noted training and seminars [54, 58, 59, 60, 62, 65, 75], colleagues’ and experts’ opinions [54, 58, 59, 62, 67], and printed materials such as textbooks [52, 54, 59, 62, 65].

### 3.8 Publication Bias

The funnel plot (Figure S2) and Egger's regression test results suggest the absence of publication bias (*z* = −0.8917, *p* = 0.3725). We have not assessed publication bias on pooled OR estimates because of the limited number of included studies.

## 4 Discussions

The present review aimed to analyze the implementation of EBP among healthcare providers in Africa. The results demonstrated that one in two healthcare providers use EBP, with the rates varying between countries and across the healthcare professions. Common factors linked to increased EBP included high knowledge of EBP, positive attitudes toward EBP, EBP training, being male providers, holding management roles, and having access to the internet and clinical guidelines. On the other hand, barriers to EBP implementation included resistance to change, poor communication, a lack of managerial support, ineffective monitoring and evaluation systems, lack of motivation, and not having EBP modules included within continuing health science professional curricula. These individual and organizational factors need to be considered while addressing the gap in evidence‐based care practices in Africa.

Although the findings mentioned above are important for policy and practice, it's notable that 92% of the participants in this review were physicians, nurses, and midwives, whereas only 8% were pharmacists, medical laboratory personnel, dentists, and radiographers. This highlights a lack of research on EBP in these underrepresented professions. Moreover, as most of the studies were conducted in Ethiopia, we may have missed certain contextual factors due to the substantial cultural and political differences across African countries.

Most of the reviewed studies did not provide detailed information on how healthcare providers are integrating evidence to inform clinical practices. For example, the subgroup analysis showed that 37% of physicians implemented EBP in their clinical decision‐making, but important information was missing, such as the specific types of physicians employing EBP (e.g., general practitioners, gynecologists, surgeons, internists, pediatricians, and urologists), the medical conditions addressed (e.g., diabetes, hypertension, and cancer), the types of evidence utilized, and the frequency of EBP implementation (e.g., daily, weekly, and monthly). Similarly, although 57% of nurses were found to implement EBP, the studies did not explicitly indicate to which nursing practices EBP had been applied (e.g., administering medications and wound care) and how consistently nurses were implementing EBP into their practices. Moreover, none of the included studies reported the effects of evidence implemented by various healthcare providers on patient outcomes.

## 5 Conclusions

This systematic review highlights the limited information available to determine the implementation of EBP among healthcare providers in Africa. Thus, further research is needed to investigate how various healthcare providers are implementing EBM to inform clinical practice and clinical outcomes. This can be achieved through more robust research designs, which examine the medical conditions addressed within providers’ scopes of practice, the types of evidence utilized, and the frequency and consistency of EBP implementation, as well as its effect on enhancing patient outcomes.

## Consent

The authors have nothing to report.

## Conflicts of Interest

The authors declare no conflicts of interest.

## Supporting information



Supporting Information

## Data Availability

All data cited in this review came from published papers and are therefore already available. The data created as part of the review are included in this published article and its Supporting Information section.
