# Supplementary material for: Limited Evidence to Fully Determine the Implementation of Evidence‐Based Practice by Healthcare Providers in Africa: A Systematic Review and Meta‐Analysis
Source: J Evid Based Med. 2025 May 15;18(2):e70032. doi: 10.1111/jebm.70032 (PMC12081785; doi:10.1111/jebm.70032)

Supplementary File

**Supplemental Table 1.** PRISMA 2009 Checklist

| **Section/topic** | **#** | **Checklist item** | **Reported on page #** |
| --- | --- | --- | --- |
| **TITLE** | | |  |
| Title | 1 | Identify the report as a systematic review, meta-analysis, or both. | 1 |
| **ABSTRACT** | | |  |
| Structured summary | 2 | Provide a structured summary including, as applicable: background; objectives; data sources; study eligibility criteria, participants, and interventions; study appraisal and synthesis methods; results; limitations; conclusions and implications of key findings; systematic review registration number. | 2 |
| **INTRODUCTION** | | |  |
| Rationale | 3 | Describe the rationale for the review in the context of what is already known. | 3-4 |
| Objectives | 4 | Provide an explicit statement of questions being addressed with reference to participants, interventions, comparisons, outcomes, and study design (PICOS). | 4 |
| **METHODS** | | |  |
| Protocol and registration | 5 | Indicate if a review protocol exists, if and where it can be accessed (e.g., Web address), and, if available, provide registration information including registration number. | Registered on Prospero with registration number [CRD42024517704] |
| Eligibility criteria | 6 | Specify study characteristics (e.g., PICOS, length of follow-up) and report characteristics (e.g., years considered, language, publication status) used as criteria for eligibility, giving rationale. | 5 |
| Information sources | 7 | Describe all information sources (e.g., databases with dates of coverage, contact with study authors to identify additional studies) in the search and date last searched. | 5 & supplementary table 2 |
| Search | 8 | Present full electronic search strategy for at least one database, including any limits used, such that it could be repeated. | 5 & supplementary table 2 |
| Study selection | 9 | State the process for selecting studies (i.e., screening, eligibility, included in systematic review, and, if applicable, included in the meta-analysis). | 5-6 |
| Data collection process | 10 | Describe method of data extraction from reports (e.g., piloted forms, independently, in duplicate) and any processes for obtaining and confirming data from investigators. | 8 & Figure 1 |
| Data items | 11 | List and define all variables for which data were sought (e.g., PICOS, funding sources) and any assumptions and simplifications made. | 5-6 |
| Risk of bias in individual studies | 12 | Describe methods used for assessing risk of bias of individual studies (including specification of whether this was done at the study or outcome level), and how this information is to be used in any data synthesis. | 6 & Supplementary Table 3 |
| Summary measures | 13 | State the principal summary measures (e.g., risk ratio, difference in means). | Proportion and odds ratio |
| Synthesis of results | 14 | Describe the methods of handling data and combining results of studies, if done, including measures of consistency (e.g., I^2^) for each meta-analysis. | 6-7 |

Page 1 of 2

| **Section/topic** | **#** | **Checklist item** | **Reported on page #** |
| --- | --- | --- | --- |
| Risk of bias across studies | 15 | Specify any assessment of risk of bias that may affect the cumulative evidence (e.g., publication bias, selective reporting within studies). | 7 |
| Additional analyses | 16 | Describe methods of additional analyses (e.g., sensitivity or subgroup analyses, meta-regression), if done, indicating which were pre-specified. | 7 |
| **RESULTS** | | |  |
| Study selection | 17 | Give numbers of studies screened, assessed for eligibility, and included in the review, with reasons for exclusions at each stage, ideally with a flow diagram. | 8 & figure 1 |
| Study characteristics | 18 | For each study, present characteristics for which data were extracted (e.g., study size, PICOS, follow-up period) and provide the citations. | 8 & Supplementary Table 4 |
| Risk of bias within studies | 19 | Present data on risk of bias of each study and, if available, any outcome level assessment (see item 12). | 13 & Supplementary Figure 2 |
| Results of individual studies | 20 | For all outcomes considered (benefits or harms), present, for each study: (a) simple summary data for each intervention group (b) effect estimates and confidence intervals, ideally with a forest plot. | 9-12 & Supplementary files |
| Synthesis of results | 21 | Present results of each meta-analysis done, including confidence intervals and measures of consistency. | 9-12 |
| Risk of bias across studies | 22 | Present results of any assessment of risk of bias across studies (see Item 15). | 13 + Supplementary Figure 2 |
| Additional analysis | 23 | Give results of additional analyses, if done (e.g., sensitivity or subgroup analyses, meta-regression [see Item 16]). | 9-10 & Table 1 |
| **DISCUSSION** | | |  |
| Summary of evidence | 24 | Summarize the main findings including the strength of evidence for each main outcome; consider their relevance to key groups (e.g., healthcare providers, users, and policy makers). | 13 |
| Limitations | 25 | Discuss limitations at study and outcome level (e.g., risk of bias), and at review-level (e.g., incomplete retrieval of identified research, reporting bias). | 13 |
| Conclusions | 26 | Provide a general interpretation of the results in the context of other evidence, and implications for future research. | 14 |
| **FUNDING** | | |  |
| Funding | 27 | Describe sources of funding for the systematic review and other support (e.g., supply of data); role of funders for the systematic review. | 15 |

*From:*  Moher D, Liberati A, Tetzlaff J, Altman DG, The PRISMA Group (2009). Preferred Reporting Items for Systematic Reviews and Meta-Analyses: The PRISMA Statement. PLoS Med 6(7): e1000097. doi:10.1371/journal.pmed1000097 For more information, visit: **www.prisma-statement.org**.

**Supplemental Table 2.** The search strategy

| **Evidence-based practice** | | **Health care provider** | **Africa** |
| --- | --- | --- | --- |
| **PubMed** | | | |
| "evidence based practice"[MeSH] OR "evidence-based practice"[ tiab] OR "evidence based practice"[ tiab] OR "evidence-based medicine"[ tiab] OR "evidence-informed practice"[ tiab] OR "evidence-based health care"[ tiab] OR "evidence-based treatments"[ tiab] OR "evidence based intervention*"[ tiab] OR “evidence-based*”[ tiab] OR “evidence based*”[ tiab] | | "Health Personnel"[MeSH] OR "healthcare provider*"[ tiab] OR "health care professional*"[ tiab] OR "health care personnel*"[ tiab] OR "allied health personnel*"[ tiab] OR "healthcare worker*"[tiab] OR "healthcare employee*"[ tiab] OR "paramedic*"[tiab] OR "health care staff*"[ tiab] OR "medical personnel*"[tiab] OR "hospital personnel*"[tiab] OR "clinician*"[tiab] OR "physician*"[ tiab] OR "doctor*"[tiab] OR "general practitioner*"[tiab] OR "dentist*"[tiab] OR "pharmacist*"[ tiab] OR "occupational therapist*"[ tiab] OR "Health officer"[ tiab] OR "Public Health"[tiab] OR "physiotherapist*"[tiab] OR "midwife*"[tiab] OR "nurse*"[tiab] OR "laboratory worker*"[tiab] OR "anaesthetist*"[tiab] | "Africa"[Mesh] OR Africa*[tiab] OR Algeria*[tiab] OR Angola*[tiab] OR Benin*[tiab] OR Botswana*[tiab] OR Burkina Faso [tiab] OR Burundi*[tiab] OR Cape Verde*[tiab] OR Cabo Verde [tiab] OR Cameron*[tiab] OR Cameroon*[tiab] OR Chad*[tiab] OR Comoros*[tiab] OR Congo*[tiab] OR Cote d'Ivoire[tiab] OR Ivory coast [tiab] OR Djibouti*[tiab] OR Egypt*[tiab] OR Eritrea*[tiab] OR Ethiopia*[tiab] OR Gabon*[tiab] OR Gambia*[tiab] OR Ghana*[tiab] OR Guinea*[tiab] OR Kenya*[tiab] OR Lesotho*[tiab] OR Liberia*[tiab] OR Libya*[tiab] OR Madagascar*[tiab] OR Malawi*[tiab] OR Mali*[tiab] OR Maurit*[tiab] OR Morocc*[tiab] OR Mozambiqu*[tiab] OR Namibia*[tiab] OR Niger*[tiab] OR Rwanda*[tiab] OR Senegal*[tiab] OR Seychelles[tiab] OR Sierra Leone*[tiab] OR Somalia*[tiab] OR Sudan*[tiab] OR Swaziland*[tiab] OR Tanzania*[tiab] OR Togo*[tiab] OR Tunisia*[tiab] OR Uganda*[tiab] OR Zambia*[tiab] OR Zimbabwe*[tiab] |
| **Embase** | | | |
| *evidence based practice/ OR "evidence-based practice”. ab,ti. OR "evidence based practice”. ab,ti.OR "evidence-based medicine”. ab,ti. OR "evidence-informed practice”. ab,ti. OR "evidence-based health care”. ab,ti. OR "evidence-based treatments”. ab,ti. OR "evidence based intervention* “. ab,ti. OR “evidence-based*”. ti,ab. OR “evidence based*”ti,ab | | *health care personnel/ OR “healthcare provider*".ab,ti.OR "health care professional*".ab,ti. OR "health care personnel*".ab,ti. OR "allied health personnel*".ab,ti. OR "healthcare worker*".ab,ti. OR  "healthcare employee*".ab,ti. OR "paramedic*".ab,ti. OR "health care staff*".ab,ti. OR "medical personnel*".ab,ti. OR "hospital personnel*".ab,ti. OR "clinician*".ab,ti. OR "physician*".ab,ti. OR "doctor*".ab,ti. OR "general practitioner*".ab,ti. OR "dentist*".ab,ti. OR "pharmacist*".ab,ti. OR "occupational therapist*".ab,ti. OR Health officer.ab,ti. OR Public Health.ab,ti. OR "physiotherapist*".ab,ti. OR "midwife*".ab,ti. OR "nurse*".ab,ti. OR "laboratory worker*".ab,ti. OR "anaesthetist*".ab,ti. | *Africa/ or "Africa*".ti,ab. or "Algeria*".ti,ab. or "Angola*".ti,ab. or "Benin*".ab,ti. or "Botswana*".ab,ti. or "Burkina Faso".ab,ti. or "Burkina Fasso".ab,ti. or "Burundi*".ab,ti. or "Cape Verde".ab,ti. or "Cape Verdean".ab,ti. or "Cabo verde".ab,ti. or "Cameron*".ab,ti. or "Cameroon*".ab,ti. or "Chad*".ab,ti. or "Comoros*".ab,ti. or "Congo*".ab,ti. or "Cote dIvoire".ab,ti. or "Ivory coast".ab,ti. or "Djibouti*".ab,ti. or "Egypt*".ab,ti. or "Eritrea*".ab,ti. or "Ethiopia*".ab,ti. or "Gabon*".ab,ti. or "Gambia*".ab,ti. or "Ghana*".ab,ti. or "Guinea*".ab,ti. or "Kenya*".ab,ti. or "Lesotho*".ab,ti. or "Liberia*".ab,ti. or "Libya*".ab,ti. or "Madagascar*".ab,ti. or "Malawi*".ab,ti. or "Mali*".ab,ti. or "Maurit*".ab,ti. or "Morocc*".ab,ti. or "Mozambiqu*".ab,ti. or "Namibia*".ab,ti. or "Niger*".ab,ti. or "Rwanda*".ab,ti. or "Senegal*".ab,ti. or "Seychelle*".ab,ti. or "Sierra Leone".ab,ti. or "Sierra Leonean".ab,ti. or "Somalia*".ab,ti. or "Sudan*".ab,ti. or "Swaziland*".ab,ti. or "Tanzania*".ab,ti. or "Togo*".ab,ti. or "Tunisia*".ab,ti. or "Uganda*".ab,ti. or "Zambia*”. ab,ti. or "Zimbabwe*".ab,ti. |
| **CINAHL** | | | |
| MH "Professional Practice, Evidence-Based" OR TI "evidence-based practice” OR AB "evidence-based practice” OR TI "evidence based practice” OR AB "evidence based practice” OR TI "evidence-based medicine” OR AB "evidence-based medicine” OR TI "evidence-informed practice” OR AB "evidence-informed practice” OR TI "evidence-based health care” OR AB "evidence-based health care” OR TI "evidence-based treatments" OR AB "evidence-based treatments" OR TI "evidence based intervention*" OR AB "evidence based intervention*" OR TI “evidence-based” OR AB “evidence-based” OR TI “evidence based” OR AB “evidence based” | MH “Health Personnel” OR TI “healthcare provider*" OR AB “healthcare provider*" OR TI "health care professional*" OR AB "health care professional*" OR TI "health care personnel*" OR AB "health care personnel*" OR TI "allied health personnel*" OR AB "allied health personnel*" OR TI "healthcare worker*" OR AB "healthcare worker*" OR TI "healthcare employee*" OR AB "healthcare employee*" OR TI "paramedic*" OR AB "paramedic*" OR TI "health care staff*" OR AB "health care staff*" OR TI "medical personnel*" OR AB "medical personnel*" OR TI "hospital personnel*" OR AB "hospital personnel*" OR TI "clinician*” OR AB "clinician*” OR TI "physician*" OR AB "physician*" OR TI "doctor*" OR AB "doctor*" OR TI "general practitioner*" OR AB "general practitioner*" OR TI "dentist*" OR AB "dentist*" OR TI "pharmacist*" OR AB "pharmacist*" OR TI "occupational therapist*" OR AB "occupational therapist*" OR TI Health officer OR AB Health officer OR TI Public Health OR AB Public Health OR TI "physiotherapist*" OR AB "physiotherapist*" OR TI "midwife*" OR AB "midwife*" OR TI "nurse*" OR AB "nurse*" OR TI "laboratory worker*" OR AB "laboratory worker*"OR TI "anaesthetist*" OR AB "anaesthetist*" | | MH Africa OR TI Africa* OR AB Africa* OR TI Algeria* OR AB Algeria* OR TI Angola*OR AB Angola* OR TI Benin*OR AB Benin* OR TI Botswana* OR AB Botswana OR TI “Burkina Faso*” OR AB “Burkina Faso*” OR TI Burundi* OR AB Burundi* OR TI “Cape Verde*” OR AB “Cape Verde*” OR TI Cameron* OR AB Cameron*OR TI Cameroon*OR AB Cameroon* OR TI Chad* OR AB chad* OR TI Comoros* OR AB Comoros* OR TI Congo* OR AB Congo* OR TI “Cote d'Ivoire” OR AB “Cote d'Ivoire” OR TI “Ivory coast” OR AB “Ivory coast” OR TI Djibouti* OR AB Djibouti* OR TI Egypt* OR AB Egypt* OR TI Eritrea* OR AB Eritrea* OR TI Ethiopia* OR AB Ethiopia* OR TI Gabon* OR AB Gabon* OR TI Gambia*OR AB Gambia* OR TI Ghana* OR AB Ghana OR TI Guinea* OR AB Guinea* OR TI Kenya* OR AB Kenya* OR TI Lesotho* OR Lesotho* OR TI Liberia* OR AB Liberia OR TI Libya* AB Libya* OR TI Madagascar* OR AB Madagascar* OR TI Malawi* OR AB Malawi* OR TI Mali* OR AB Mali* OR TI Maurit* OR AB Maurit* OR TI Morocc* OR AB Morocc* OR TI Mozambiqu* OR AB Mozambiqu* OR TI Namibia* OR AB Namibia* OR TI Niger* OR AB Niger* OR TI Rwanda* OR AB Rwanda* OR TI Senegal* OR AB Senegal* OR TI Seychelles* OR TI Seychelles* OR TI “Sierra Leone*” OR AB “Sierra Leone*” OR TI Somalia* OR AB Somalia” OR TI Sudan* OR AB Sudan* OR TI Swaziland* OR AB Swaziland* OR TI Tanzania* OR AB Tanzania* OR TI Togo* OR AB Togo* OR TI Tunisia* OR AB Tunisia OR TI Uganda* OR AB Uganda* OR TI Zambia* OR TI Zambia* OR TI Zimbabwe* OR AB Zimbabwe* |
| **Scopus** | | | |
| (TITLE-ABS ("evidence-based practice" OR "evidence based practice" OR "evidence-based medicine" OR "evidence-informed practice" OR "evidence-based health care" OR "evidence-based treatments" OR "evidence based intervention*" OR “evidence-based” OR “evidence based”)) | (TITLE-ABS ("healthcare provider*" OR "health care professional*" OR "health care personnel*" OR "allied health personnel*" OR "healthcare worker*" OR "healthcare employee*" OR "paramedic*" OR "Health care staff*" OR "Medical personnel*" OR "hospital personnel*" OR clinician* OR physician* OR doctor* OR"general practitioner*" OR dentist* OR pharmacist* OR "occupational therapist*" OR "Health office" OR "Public Health" OR physiotherapist* OR midwife* OR nurse* OR "laboratory worker*", OR anaesthetist*)) | | (TITLE-ABS (Africa OR Algeria* OR Angola* OR Benin* OR Botswana* OR "Burkina Faso" OR Burundi* OR "Cape Verde*" OR "Cabo Verde" OR Cameron* OR Cameroon* OR Chad* OR Comoros* OR Congo* OR "Cote d'Ivoire" OR "Ivory coast" OR Djibouti* OR Egypt* OR Eritrea* OR Ethiopia* OR Gabon* OR Gambia* OR Ghana* OR Guinea* OR Kenya* OR Lesotho* OR Liberia* OR Libya* OR Madagascar* OR Malawi* OR Mali* OR Maurit* OR Morocc* OR Mozambiqu* OR Namibia* OR Niger* OR Rwanda* OR Senegal* OR Seychelles OR "Sierra Leone*" OR Somalia* OR Sudan* OR Swaziland* OR Tanzania* OR Togo* OR Tunisia* OR Uganda* OR Zambia* OR Zimbabwe*)) |
| **Global Index Medicus (GIM)** | | | |
| tw: (evidence-based practice) OR (evidence based practice) OR (evidence-based medicine) OR (evidence-informed practice) OR (evidence-based health care) OR (evidence-based treatments) OR (evidence-based intervention*) OR (evidence-based) OR (evidence based) | tw: (healthcare provider*) OR (health care professional*) OR (health care personnel*) OR (allied health personnel*) OR (healthcare worker*) OR (healthcare employee*) OR (paramedic*) OR (Health care staff*) OR (Medical personnel*) OR (hospital personnel*) OR (clinician*) OR (physician*) OR (doctor*) OR (general practitioner*) OR (dentist*) OR (pharmacist*) OR (occupational therapist*) OR (Health officer) OR (Public Health) OR (physiotherapist*) OR (midwife*) OR (nurse*) OR (laboratory worker*) OR (anaesthetist*) | | tw: (Africa) OR (Algeria*) OR (Angola*) OR (Benin*) OR (Botswana*) OR (Burkina Faso) OR (Burundi*) OR (Cape Verde*) OR (Cabo Verde) OR (Cameron*) OR (Cameroon*) OR (Chad*) OR (Comoros*) OR (Congo*) OR (Cote d'Ivoire) OR (Ivory coast) OR (Djibouti*) OR (Egypt*) OR (Eritrea*) OR (Ethiopia*) OR (Gabon*) OR (Gambia*) OR (Ghana*) OR (Guinea*) OR (Kenya*) OR (Lesotho*) OR (Liberia*) OR (Libya*) OR (Madagascar*) OR (Malawi*) OR (Mali*) OR (Maurit*) OR (Morocc*) OR (Mozambiqu*) OR (Namibia*) OR (Niger*) OR (Rwanda*) OR (Senegal*) OR (Seychelles) OR (Sierra Leone*) OR (Somalia*) OR (Sudan*) OR (Swaziland*) OR (Tanzania*) OR (Togo*) OR (Tunisia*) OR (Uganda*) OR (Zambia*) OR (Zimbabwe*) |
| **Total: 7,713** |  | |  |

Date limiter: Date: 01/01/1992; language: English and Articles done on human. Date of search: March 05, 2024

**Supplemental Table 3:** Methodological quality assessment from the Mixed Methods Appraisal Tool (MMAT)

|  | **1** | **2** | **3** | **4** | **5** | **6** | **7** | **The overall quality of the study** |
| --- | --- | --- | --- | --- | --- | --- | --- | --- |
| **Quantitative study** |  |  |  |  |  |  |  |  |
| Sendekie AD, 2022 | Y | Y | Y | N | Y | Y | Y | 6 |
| Kadri G, 2022 | Y | U | Y | Y | U | N | U | 3 |
| Shibabaw AA, 2023 | Y | N | Y | N | Y | Y | Y | 5 |
| Mortada EM 2013 | Y | U | Y | Y | Y | Y | Y | 6 |
| Oluwadiya K, 2024 | Y | U | Y | Y | Y | Y | Y | 6 |
| Megersa Y 2023 | Y | Y | Y | Y | Y | Y | Y | 7 |
| Abdel-Kareem A 2019 | Y | Y | Y | Y | Y | Y | Y | 7 |
| Beshir MA,2017 | Y | N | Y | Y | Y | Y | Y | 6 |
| Aynalem ZB 2021 | Y | Y | Y | Y | Y | Y | Y | 7 |
| Wodajo S 2023 | Y | Y | Y | Y | Y | Y | Y | 7 |
| Ahmed HS 2015 | Y | U | N | N | U | Y | U | 2 |
| Lamesa D 2023 | Y | U | Y | Y | Y | Y | Y | 6 |
| Dessie G 2020 | Y | Y | Y | N | Y | Y | U | 6 |
| Worku T,2019 | Y | N | Y | Y | Y | Y | Y | 6 |
| Dagne AH 2021 | Y | Y | Y | Y | Y | Y | Y | 7 |
| Alemayehu A 2021 | Y | Y | Y | Y | Y | Y | Y | 7 |
| Nalweyiso D 2019 | Y | Y | Y | N | Y | N | Y | 5 |
| Taye EB 2024 | Y | Y | Y | Y | Y | Y | Y | 7 |
| Kassahun F,1017 | Y | Y | Y | Y | Y | Y | Y | 7 |
| Zeidan AZ 2010 | Y | Y | U | U | Y | Y | U | 4 |
| Wassie MA, 2018 | Y | Y | Y | Y | Y | Y | Y | 7 |
| Bankole SO, 2022 | Y | Y | U | U | U | N | Y | 3 |
| Golge AM 2024 | Y | Y | Y | Y | Y | Y | Y | 7 |
| Alene Z 2021 | Y | Y | Y | Y | Y | Y | Y | 7 |
| **Qualitative study** |  |  |  |  |  |  |  |  |
| Dagne AH 2021 | Y | Y | Y | Y | Y | Y | Y | 7 |
| Yiridomoh GY 2020 | Y | Y | Y | Y | N | Y | Y | 6 |
| **Mixed method** |  |  |  |  |  |  |  |  |
| Hadgu G 2015 | Y | U | N | Y | N | Y | Y | 4 |
| Assefa K 2021 | Y | U | Y | Y | N | Y | U | 4 |
| Unadkat MB 2021 | Y | Y | Y | Y | Y | Y | Y | 7 |
| Atakro CA,2020 | Y | Y | Y | Y | Y | Y | Y | 7 |
| Barako TD 2012 | Y | Y | Y | Y | N | Y | Y | 6 |
| Degu AB 2022 | Y | Y | Y | Y | Y | Y | Y | 7 |
| Dereje B,2019 | Y | Y | N | N | Y | N | Y | 4 |

**Screening questions for all studies:** (1) Are there clear research questions? (2) Do the collected data allow us to address the research questions?

Q**uantitative studies: i) Descriptive** q**uantitative studies:** (3) Is the sampling strategy relevant to address the research question? (4) Is the sample representative of the target population? (5) Are the measurements appropriate? (6) Is the risk of nonresponse bias low? (7) Is the statistical analysis appropriate to answer the research question? **ii) Analytical** q**uantitative studies** (3) Are the participants representative of the target population? (4) Are measurements appropriate regarding both the outcome and intervention (or exposure)? (5) Are there complete outcome data? (6) Are the confounders accounted for in the design and analysis? (7) During the study period, is the intervention administered (or exposure occurred) as intended?

**Qualitative studies**: (3) Is the qualitative approach appropriate to answer the research question? (4) Are the qualitative data collection methods adequate to address the research question? (5) Are the findings adequately derived from the data? (6) Is the interpretation of results sufficiently substantiated by data? (7) Is there coherence between qualitative data sources, collection, analysis, and interpretation?

**Mixed methods studies:** (3) Is there an adequate rationale for using a mixed methods design to address the research question? (4) Are the different components of the study effectively integrated to answer the research question? (5) Are the outputs of the integration of qualitative and quantitative components adequately interpreted? (6) Are divergences and inconsistencies between quantitative and qualitative results adequately addressed? (7) Do the different components of the study adhere to the quality criteria of each tradition of the methods involved?

*Each item was rated “Y = Yes”, “N = No”, or “U = Unclear” was awarded where not enough information was provided. High quality: meets ≥ 7 criteria, Moderate quality: meets ≥ 4 criteria, Low quality: < 4 criteria.*

**Supplementary Table 4.** Characteristics of studies included in this review (n = 33).

| **First author, publication year and reference** | **Country and study year** | **Study Population**  **and sample size** | **Facility type** | **Type of Study** | **Key findings** |
| --- | --- | --- | --- | --- | --- |
| Shibabaw AA,2023(68) | Ethiopia, 2022 | Physician, Nurse, Midwifery,  Laboratory, Pharmacy(n=409) | Hospital | Quantitative | Prevalence of EBP was 36.2%. The factors include being a male provider (AOR=1.85), having EBP training (AOR = 5 43), good knowledge (AOR = 1 91), a favourable attitude (AOR = 1 91), and >5 years work experience (AOR = 1 58) |
| Kadri G, 2022(69) | Morocco, 2021 | Dentists(n=209) | Public and Private sector | Quantitative | EBD training (β=0.63), knowledge of PICO question formulation: (β=0.90) and the knowledge of relevant information sources such as the Cochrane library (β = 0.44). |
| Barako TD, 2012(57) | Kenya | Nurses (n=130 for quantitative, n=14 for qualitative) | Hospital | Mixed methods | Factors were a lack of confidence to critically review EBP literature (AOR=0.06) and negative attitudes towards EBP (AOR=0.49). *From the qualitative analysis*: staff shortage, time constraints, absence of guidelines, absence of monitoring and evaluation. |
| Mortada EM, 2013(67) | Egypt, 2012 | Physicians(n=184) | Hospital | Quantitative | The proportion of EBM users was 37.5%. |
| Sendekie AD,2022(70) | Ethiopia, 2021 | Medical doctors, Midwives, and IESO(n=391) | Hospital | Quantitative | The magnitude of evidence-based care was 54.7%. Good knowledge [AOR = 2.1], computer access [AOR = 2.04], >=5 years of work experience [AOR= 2.13] and training [AOR = 1.81] were found to be factors. |
| Oluwadiya K, 2024(55) | Nigeria, 2019 | Surgeons(n=169) | Hospital | Quantitative | A total of 72.1% of participants reported that they have used EBM in their practice. |
| Atakro CA, 2020(65) | Ghana, 2017 | Nurses (n=102 for quantitative, n=20 for qualitative) | Hospital | Mixed methods | *From qualitative analysis*: increased workloads, time shortage, lack of EBP training, lack of workshop, inadequate guidelines, lack of library and internet facilities. |
| Megersa Y, 2023(60) | Ethiopia, 2021 | Nurses (n=403) | Hospital | Quantitative | EBP utilisation was 52.4%. current management role (AOR=2.7), diploma educational level (AOR=0.353), good knowledge (AOR=1.785), primary hospital (AOR=0.456), availability of time (AOR=0.523), and cooperative and supportive colleagues (AOR=0.429) were associated with good utilisation of EBP. |
| Abdel-Kareem A, 2019(71) | Egypt, 2017 | Physicians(n=398) | Hospital | Quantitative | EBP was 8.7 %. |
| Beshir MA,2017(72) | Ethiopia 2015 | Physicians, Nurses, Midwifery, Laboratory technicians, Health officers, psychiatrists, radiologists, physiotherapists, and pharmacists (n=431) | Hospital | Quantitative | EBP utilization was 53%. Significant factors include good knowledge (AOR=1.612, internet access (AOR=1.831), training (AOR=1.906), and availability of time (AOR=1.698). |
| Aynalem ZB, 2021(56) | Ethiopia 2019 | Nurses (n=671), | Hospital | Quantitative | EBP utilization was 55%. Predicting factors were being single marital status (AOR = 1.662), work experience of <5 years (AOR = 1.849), good knowledge (AOR = 2.044), effective nurse-patient communication (AOR = 2.537), EBP training (AOR = 3.224), internet access (AOR = 1.655) and availability of guideline (AOR = 1.827) |
| Dagne AH, 2021(35) | Ethiopia  2019-2020 | Nurses, midwives (n=86) | Hospital | Qualitative | Knowledge, skill and attitude gap, increased workloads and time shortage, lack of EBP training or workshop, lack of motivation/interest, lack of supervisor/manager support, inadequate resources (staff, computers, guidelines, lack of workplace library or internet access, absence of monitoring and evaluation, insufficient research articles/literature |
| Wodajo S 2023(73) | Ethiopia 2022 | Physicians, Midwife, IESO (n=278) | Hospital | Quantitative | The magnitude of evidence-based care was 63.7%. Factors were having MSc and above (AOR=5.75), good knowledge (AOR=2.95), positive attitude (AOR=3.13), more than 5 obstetric care providers per shift (AOR=2.31), less than 10 deliveries within a day (AOR=4.61), and availability of guidelines (AOR=2.88). |
| Yiridomoh GY 2020(74) | Ghana 2019 | Nurses (n=13) | Hospital and  Health centres | Qualitative | Knowledge or skill gap, increased workloads and time shortage, inadequate resources (staff, computers, guidelines), lack of workplace library or internet access, lack of EBP concept in the curriculum, lack of education opportunities |
| Ahmed HS 2015(75) | Sudan 2008–2009 | Physicians (n=80) | Hospital | Quantitative | EBM practice was found to be 56.3%. |
| Lamesa D 2023(59) | Ethiopia 2022 | Nurses (n=418) | Hospital | Quantitative | The utilization of EBP was 58.1%. Being a male provider (AOR 4.65), having work experience of >5 years (AOR 6.83), being a head nurse (AOR=1.72), having master’s degree (AOR 2.78), and availability of guideline (AOR 1.24) were significantly associated with EBP. |
| Dessie G. 2020(58) | Ethiopia 2017 | Nurses, midwives, and physicians (n=405). | Hospital | Quantitative | Work experience (β=–0.94), BSc nurse (β=3.36); general practitioner (β=7.60); specialist physician (β=14.76); lack of regular orientation (β=–0.90); working in a paediatric ward: (β= –1.77) were significantly associated with EBP. |
| Degu AB 2022(63) | Ethiopia 2020 | Nurses (n=507 for quantitative, n=12 for qualitative) | Hospital | Mixed methods | EBP was associated with having MSc and above (AOR=2.15), supportive administration (AOR = 1.89), favourable attitude towards EBP (AOR = 1.80), and available information sources (AOR: 2.32). *Themes from qualitative analysis:* Knowledge or skill gap, lack of EBP training, resistance to change, lack of manager support, inadequate resources, absence of incentives, lack of workplace library or internet access, presence of established training centre, promoting EBP and experience-sharing between hospitals |
| Worku T,2019(61) | Ethiopia 2017 | Physicians (n=124) | Hospital | Quantitative | Factors include difficulty in understanding research reports (AOR = 0.72), and ability to retrieve evidence (AOR 3.15). |
| Dagne AH 2021(76) | Ethiopia 2019 | Nurses and midwives (n=790), | Hospital | Quantitative | The magnitude of EBP implementation was 34.7%. Age between 25-29 years (AOR = 5.98), positive attitude (AOR = 5.02), good knowledge (AOR = 3.06), desirable work index (AOR = 3.9) and self-efficacy (AOR = 12.5) were significant associated factors |
| Alemayehu A 2021(33) | Ethiopia 2019 | Nurses (n=671) | Hospital | Quantitative | EBP utilisation was found to be 55%. Good knowledge (AOR=2.044); EBP training (AOR=3.224); effective nurse‑patient communication (AOR= 2.537), Internet access (AOR=1.655); good staff EBP culture (AOR=1.841), presence of guidelines (AOR=1.827) were the significant predictors for utilising EBP. |
| Nalweyiso D 2019(31) | Uganda | Radiographers (n=83) |  | Quantitative | 59% of radiographers used EBP in clinical practice. |
| Taye EB 2024(54) | Ethiopia 2023 | Midwives (n=384) | Hospital | Quantitative | Implementation of EBP was 51.82%. Primary hospital [β: 4.12; 95], attending continuous professional development [β: 2.36], internet access [β: 3.68], participating in conferences [β: 4.94], and seminars [β: 4.99] were found to be significantly associated with EBP implementation |
| Kassahun F,2017(77) | Ethiopia 2015 | Physicians, Health officers, Midwives, Nurses (n=207) | Hospital | Quantitative | The proportion of EBP was 38.3%. EBP Training (AOR=4.50), good knowledge (AOR=5.30), favourable attitude (AOR=3.34) and conference participation (AOR=0.34) were factors. |
| Zeidan AZ 2010(52) | Sudan 2008 | Resident doctors (n=141) | Hospital | Quantitative | EBM use for their clinical practice was 10% |
| Dereje B, 2019(64) | Ethiopia 2018 | Nurses (n=253 for quantitative, n=12 for qualitative). | Hospital | Mixed methods | EBP utilization was 51.8%. Factors were being a male provider (AOR=2.401), being a head nurse (AOR=5.227), having a BSc (AOR=3.186), good knowledge (AOR=2.084), ability to properly interpret the results of research (AOR= 4.282), being teaching hospital (AOR=4.798) and autonomy to change practice (AOR=2.590). *Themes from qualitative analysis: r*esistance to change, poor communication, lack of motivation, lack of manager support, absence of incentives, lack of workplace library or internet access, insufficient research articles |
| Wassie MA, 2018(66) | Ethiopia 2014 | Medical laboratory (n=169) | Hospital | Quantitative | The level of EBP was 40.8%. good knowledge (AOR=2.22), internet access (AOR=2.43), organizational adoption of EBP (AOR=2.79) and being single in marital status (AOR=2.21) were found to be factors associated with EBP |
| Bankole SO, 2022(78) | Nigeria 2019 | Nurses (n=305) | Hospital | Quantitative | The utilisation of EBP among the nurses was 78.7%. |
| Golge AM 2024(53) | Ethiopia 2022 | Nurses (n=385) | Hospital | Quantitative | The implementation of EBP was 54.5%. attitude (β: 0.448); self-efficacy (β: 0.528); being a diploma nurse (β: -3.925); knowledge (β: 1.213); working in a referral hospital (β: 3.513); availability of job rotation (β: 3.475) were found to be significant factors |
| Alene Z, 2021(79) | Ethiopia 2020 | Nurses (n=135) | Hospital | Quantitative | The utilization of EBP was 85.2% |
| Hadgu G, 2015(80) | Ethiopia 2014 | Nurses (n=210) | Hospital | Mixed method | EBP utilization was 15.7%. Age of 30-34 years (AOR=9.47), good knowledge (AOR= 3.2), having free time (AOR=7.9), having supportive managers (AOR=5) and skill (AOR=2.4) have a significant association with EBP |
| Assefa K 2021(81) | Ethiopia 2020 | Nurses (n=422) | hospital | Mixed method | Implementation of EBP was 58%. Increased workloads and time shortage, lack of EBP training or workshop, resistance to change, poor communication, lack of supervisor/manager support, inadequate resources, absence of incentives and rewards |
| Unadkat MB 2021(32) | Kenya 2017 | Resident physicians (n=101) | Hospital | Mixed method | EBM utilization was 65%. *Theme*: Increased workloads and time shortage |

*β: beta coefficient, AOR= Adjusted Odds Ratio, IESO=Integrated Emergency Surgical Officers,*

**Supplemental Figure 1.** Proportion of healthcare providers with A) good knowledge and B) positive attitude towards EBP


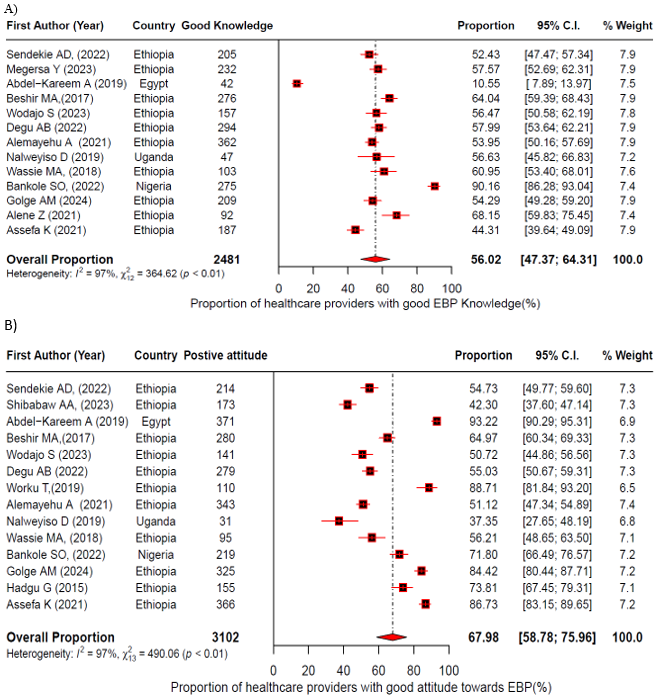


**Supplementary Table 5:** A Summary Qualitative Synthesis of Factors to EBP in Africa

| Study references | Atakro CA,  2020 (65) | Dagne AH, 2021 (35) | Barako TD, 2012 (57) | Degu AB, 2022 (63) | Yiridomoh GY,2020 (74) | Dereje B, 2019 (64) | Assefa K, 2021 (81) | Unadkat MB, 2021 (32) | Total |
| --- | --- | --- | --- | --- | --- | --- | --- | --- | --- |
| **Healthcare providers factors** |  |  |  |  |  |  |  |  |  |
| Knowledge or skill gap |  |  |  |  |  |  |  |  | 4 |
| Increased workloads and time shortage |  |  |  |  |  |  |  |  | 6 |
| Lack of EBP training or workshop |  |  |  |  |  |  |  |  | 4 |
| Resistance to change |  |  |  |  |  |  |  |  | 3 |
| Poor communication |  |  |  |  |  |  |  |  | 2 |
| Lack of motivation/interest |  |  |  |  |  |  |  |  | 2 |
| **Healthcare organization factors** |  |  |  |  |  |  |  |  |  |
| Lack of supervisor/manager support |  |  |  |  |  |  |  |  | 4 |
| Inadequate resources (staff, computers,  guidelines) |  |  |  |  |  |  |  |  | 6 |
| absence of incentives and rewards |  |  |  |  |  |  |  |  | 3 |
| Lack of workplace library or internet  access |  |  |  |  |  |  |  |  | 6 |
| Established training centre* |  |  |  |  |  |  |  |  | 1 |
| Promoting EBP and experience-sharing  between hospitals* |  |  |  |  |  |  |  |  | 1 |
| Absence of monitoring and evaluation |  |  |  |  |  |  |  |  | 2 |
| **Educational factors** |  |  |  |  |  |  |  |  |  |
| Lack of EBP concept in the curriculum |  |  |  |  |  |  |  |  | 1 |
| Lack of education opportunities |  |  |  |  |  |  |  |  | 1 |
| **Evidence related factors** |  |  |  |  |  |  |  |  |  |
| Insufficient research articles/literature |  |  |  |  |  |  |  |  | 4 |

*^*^Facilitating factors for EBP*

**Supplementary Figure 2**: Funnel plot for EBP among healthcare factors.


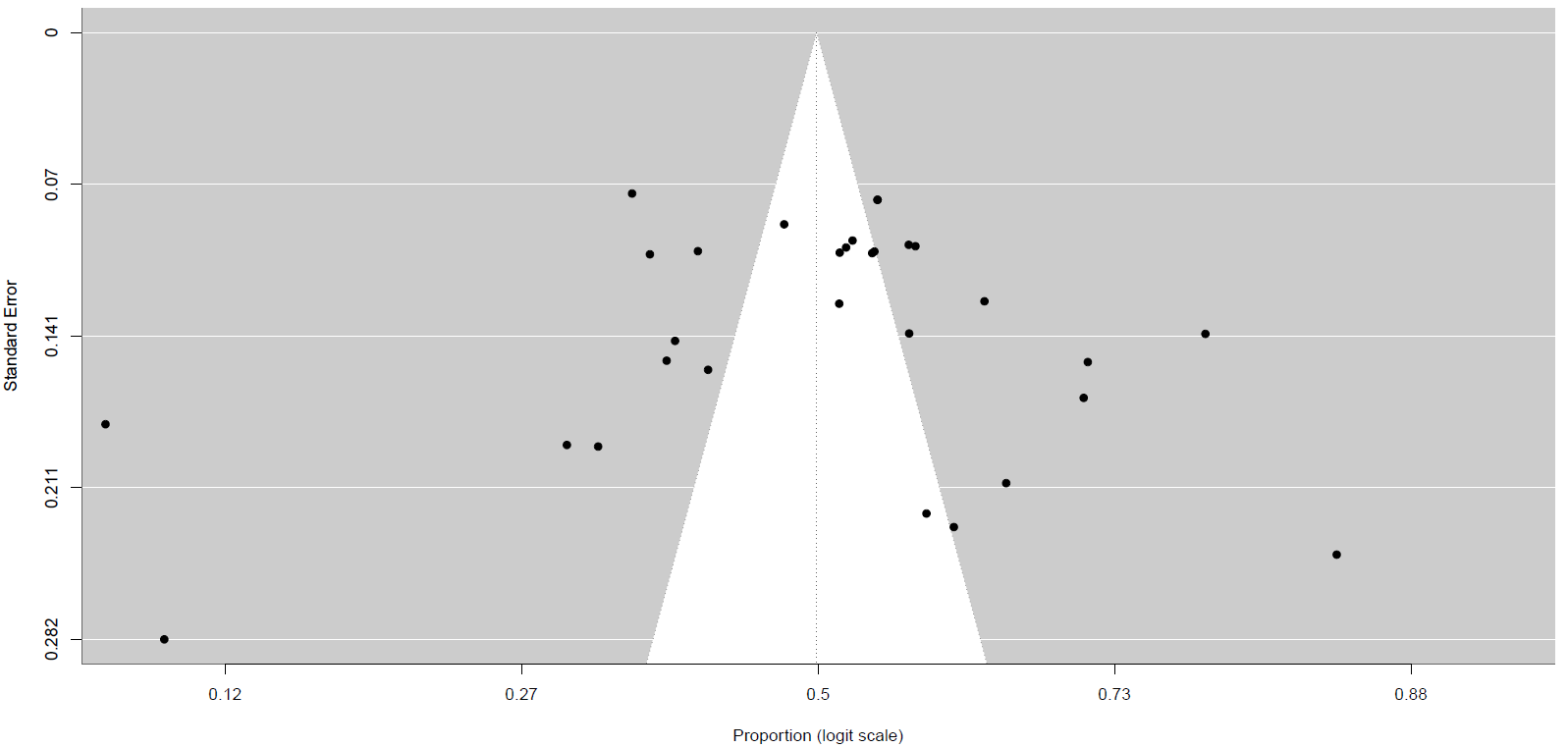

Supplement: Supplementary file 1 — Supporting Information [file JEBM-18-0-s001.docx]
